# The regulation and interaction of colon cancer‐associated transcript‐1 and miR7‐5p contribute to the inhibition of SP1 expression by solamargine in human nasopharyngeal carcinoma cells

**DOI:** 10.1002/ptr.6555

**Published:** 2019-12-10

**Authors:** JingJing Wu, XiaoJuan Tang, ChangJu Ma, Yao Shi, WanYin Wu, Swei Sunny Hann

**Affiliations:** ^1^ Laboratory of Tumor Biology, Department of Medical Oncology Guangdong Provincial Hospital of Chinese Medicine, The Second Clinical College of Guangzhou University of Chinese Medicine Guangzhou China; ^2^ Department of Cerebrovascular Disease Guangdong Provincial Hospital of Chinese Medicine, The Second Clinical College of Guangzhou University of Chinese Medicine Guangzhou China

**Keywords:** CCAT1, miR7‐5p, Nasopharyngeal carcinoma cells, solamargine, SP1

## Abstract

Nasopharyngeal carcinoma (NPC) is a common head and neck malignancy with higher incidence in Southern China and Southeast Asia. Solamargine (SM), a steroidal alkaloid glycoside, has been shown to have anticancer properties. However, the underlying mechanism involved remains undetermined. In this study, we showed that SM inhibited the growth of NPC cells. Mechanistically, we found that solamargine decreased lncRNA colon cancer‐associated transcript‐1 (CCAT1) and increased miR7‐5p expression. There was a reciprocal interaction of CCAT1 and miR7‐5p. In addition, SM inhibited the expression of SP1 protein and promoter activity, which was strengthened by miR7‐5p mimics and inhibited by overexpressed CCAT1. MiR7‐5p could bind to 3'‐UTR of SP1 and attenuated SP1 gene expression. Exogenously expressed SP1 feedback resisted SM‐increased miR7‐5p expression and more importantly reversed SM‐inhibited growth of NPC cells. Finally, SM inhibited NPC tumor growth in vivo. Collectively, our results show that SM inhibits the growth of NPC cells through reciprocal regulation of CCAT1 and miR7‐5p, followed by inhibition of SP1 gene expression in vitro and in vivo. The interregulation and correlation among CCAT1, miR7‐5p and SP1, and the feedback regulatory loop unveil the novel molecular mechanism underlying the overall responses of SM in anti‐NPC.

**Abbreviations**
EdU: (5‐ethynyl‐2'‐deoxyuridine)
SP1: (specific protein 1)
MTT: [3‐(4,5‐dimethylthiazol‐2‐yl)‐2,5‐diphenyltetrazolium bromide]
micro RNA‐7‐5p: (miR‐7‐5p)
Dimethyl sulfoxide: (DMSO)
Dulbecco's Modified Eagle Medium: (DMEM)
fetal bovine serum: (FBS)
sodium dodecyl sulfate: (SDS)
horseradish peroxidase: (HRP)
polymerase chain reaction: (PCR)
glyceraldehyde‐3‐phosphate dehydrogenase: (GAPDH)
cycle threshold: (CT)
half‐limiting dose: (IC50)
Long non‐coding RNA: (lncRNA)
3'untranslated region: (3'UTR)
epithelial‐mesenchymal transition: (EMT)
zinc finger NFX1‐type containing 1: (ZNFX1)
wild type: (WT)

## 1 INTRODUCTION

Nasopharyngeal carcinoma (NPC) is a common malignancy in the southern China, Southeast Asia, and North Africa (Lee, Ma, Ng & Chan, 2015). The genetic susceptibility, environmental factors, exposure to chemical carcinogens, and Epstein–Barr virus (EBV) infection are considered the major reasons for this cancer type (Hildesheim & Wang, 2012). Radiotherapy alone or concurrent chemoradiotherapy are the main treatments for NPC patients and the survival rate has improved significantly especially in patients with locally and regionally advanced NPC (Baujat et al., 2006; Lee, Ma, Ng & Chan, 2015; Li et al., 2015). However, radiotherapy for NPC is notoriously difficult due to the invasive characteristics of the tumor, early local and distant metastasis; concomitant chemoradiotherapy‐related toxicities (Du, Ying, Kong, Zhai & Hu, 2015; Jiang et al., 2015). Thus, better understanding of the molecular mechanisms underlying occurrence and progression of NPC is essential for the early diagnosis and the development of effective therapeutic agents. Therefore, search for the novel therapeutic modality to increase the therapeutic efficacy are urgently needed.

Natural phytochemicals derived from medicinal plants have been considered an ideal approach in prevention and treatment of cancer. Solamargine (SM), a major steroidal alkaloid glycoside came from herb *Solanum nigrum* L, have shown to suppress tumor growth and induce apoptosis in the several types of cancers (Cui, Wen, Cui, Gao, Sun & Lou, 2012; Friedman, 2015; Munari et al., 2014; Zhou et al., 2014). *Solanum incanum* extract (SR‐T100), which contains SM as major active ingredient, showed to inhibit growth of ovarian cancer cells in vitro and in vivo through downregulation of the some stem cell markers, such as aldehyde dehydrogenase 1, transcription factor CCAAT‐enhancer‐binding protein β (C/EBP β), among others (Wu, Chiu, Young, Chang, Huang & Chou, 2015). We previously demonstrated that SM inhibited the growth of human lung cancer cells through inactivation of phosphatidylinositol 3‐kinase (PI3‐K)/Akt signaling pathway and reduction of transcription factor SP1 and p65 proteins. This resulted in the inhibition of prostaglandin E2 receptor EP4 gene expression (Chen et al., 2015). However, limited data have been found for the links of SM to the NPC. The mechanisms and potential nontoxic benefits by which this agent controls NPC cell growth remain unknown.

Long noncoding RNA (lncRNA) are a class of regulatory noncoding RNAs with over 200 nucleotides in length. Deregulation of lncRNAs has been observed in a variety of human diseases, including cancer (Gao L, 2013; T.R. Mercer, 2009) and associated with clinic‐pathological parameters, including proliferation, metastasis, recurrence, and overall survival (Bhan, Soleimani & Mandal, 2017). Among these, colon cancer‐associated transcript‐1 (CCAT1), which was first identified in colon cancer with a length of 2,628 nucleotides and mapped to chromosome 8q24.2, has been found to be highly expressed in multiple types of cancer and played a critical role in various biological processes, such as proliferation, invasion, migration, drug resistance, and survival (Guo & Hua, 2017; Shan T, 2017). This finding renders CCAT1 attractive as target for therapeutic intervention in cancer. In NPC cells, CCAT1 was highly expressed in NPC tissues compared with normal nasopharyngeal epithelial ones and silencing of CCAT1 inhibited growth, migration, and invasion in NPC cells (Dong, Yuan & Jin, 2018). Conversely, increased CCAT1 expression resulted in significantly enhancing paclitaxel resistance in NPC cells. Moreover, bioinformatics analysis, luciferase reporter, and RIP experiments indicated that the induced CCAT1 sponged miR‐181a and miR‐181a could directly bind to CCAT1 mRNA in NPC cells (Wang, Zhang & Hao, 2017). At present, the function role of CCAT1 and mechanism underlying CCAT1‐mediated cancer development and progression still remain to be determined.

As single stranded noncoding RNA molecules, miRNAs have been reported to control cellular and physiological processes, such as tumorigenesis, progression, metastasis, and angiogenesis, via regulating the expression of protein‐coding genes by repressing translation or cleaving RNA transcripts in a sequence‐specific manner (Tutar, 2014). The ability of miRNAs to target multiple genes in cancer biology makes them promising target for the development of potential biomarkers of cancer that could potentially contribute to diagnosis, progression, and treatment strategies (Takahashi, Prieto‐Vila, Kohama & Ochiya, 2019; Tang, Wang & Hann, 2019). MiR7‐5p is mainly considered as a tumor suppressor miRNA that inhibits tumor growth via regulating multiple oncogenic signal pathways (Dong, Xie & Xu, 2019; Hu et al., 2019; Jia et al., 2019). LncRNA FOXD2 adjacent opposite strand RNA 1 acted as a competitive endogenous RNA for miR7‐5p, showed to increase the expression of telomerase reverse transcriptase, which further promoted the cancer stem cells features and anoikis resistance in thyroid cancer cells (Liu et al., 2019). Bioinformatic analysis indicated that poly ADP‐ribose polymerase 1 (PARP1) was a direct target of miR7‐5p, and PARP1 expression was inhibited by miR7‐5p in small cell lung cancer cells. This resulted in sensitizing cancer cells to doxorubicin suggesting that the regulation of miR7‐5p could be potential for overcoming chemoresistance in lung cancer (Lai, Yang, Zhu, Ruan, Huang & Zhang, 2019). Regardless, the potential mechanism underlying the regulation of miR7‐5p involved in the tumorigenesis and progression of NPC remains unknown.

SP1, a well‐known transcription factor, is implicated in an ample variety of essential biological processes, such as cell growth, differentiation, apoptosis, and carcinogenesis via activating the transcription of many cellular genes that contain putative CG‐rich SP‐binding sites in their promoters. Of note, there was limited information for the correlations of CCAT1, miR7‐5p, and SP1, although SP1 has been reported to regulate many other lncRNA and/or miRNAs in cancer cells (Jin, Zhang, Hu, Zhang, Guo & Feng, 2019; Li et al., 2019; Li, Qiu, Sun, Zhang & Wang, 2019; Ren, Zhang & Jiang, 2018). Thus, the mechanisms underlying the connections among these molecules remain to be determined.

In this study, we explored the novel mechanism by which SM controlled growth of human NPC cells. Our results indicated that SM inhibited the growth of NPC cells through reciprocal regulation of CCAT1 and miR7‐5p, followed by inhibition of SP1 gene expression in vitro and in vivo.

## 2 MATERIALS AND METHODS

### 2.1 Reagents

Monoclonal antibodies against SP1 and β‐actin were obtained from Cell Signaling Technology Inc. (Beverly, MA, USA). MTT powder was purchased from Sigma‐Aldrich (St. Louis, MO, USA). Control, SP1 (pCMV6‐SP1), and CCAT1 overexpression plasmids (MO2‐CCAT1) and SP1 promoter constructs and internal control secreted alkaline phosphatase were purchased from GeneCopoeia (Rockville, MD, USA). Cell‐Light^TM^ EdU DNA cell proliferation kit and miR7‐5p mimics were obtained from Ribo Biological Co., Ltd. (Guangzhou, China). SM was purchased from Chengdu Must Bio‐technology Company (Chengdu, Sichuan, China) and was dissolved with DMSO as stock solution and freshly diluted to the working concentration with medium before use.

### 2.2 Cell culture and chemicals

The NPC cell lines HNE2 and C666‐1 were obtained from the (Sun Yat‐sen Memorial Hospital, Sun Yat‐sen University, Guangzhou, Guangdong Province, China). All cell lines have been tested and authenticated for absence of mycoplasma, genotypes, drug response, and morphology in the Laboratory. C666‐1 is commonly studied EBV‐positive and undifferentiated NPC cells. HNE2 is EBV‐negative and poorly differentiated NPC cell line. Cells were grown in F12K or DMEM (1:1) medium (obtained from GIBCO, Life Technologies, Grand Island, NY, USA) with supplemented 10% fetal bovine serum. Lipofectamine 3000 reagent was purchased from Invitrogen (Shanghai, China).

### 2.3 Cell viability assay

Cell viability was evaluated by the MTT [3‐(4,5‐dimethyl‐2‐thiazolyl)‐2,5‐diphenyl‐2H‐tetrazolium bromide] assay as described previously (Zhao et al., 2015). In brief, NPC cells were counted and seeded in a 96‐well microtitre plate (1 × 10^4^ cells/96‐microwell plate). The cells were treated with increasing concentrations of SM for up to 72 hr. After incubation, 20‐μl MTT solutions (5 g/L) were added to each well, and cancer cells were incubated at 37°C for additional 4 hr. The resulting blue formazan product was dissolved in dimethyl sulfoxide and the absorbance of the sample was measured by ELISA reader (BioTek, Epoch, VT, USA) to determine the absorbance at 570 nm. Cell viability (%) was calculated as follows: (absorbance of test sample/absorbance of control) × 100%.

### 2.4 EdU incorporation assay

Cell proliferation was determined by Cell‐Light^TM^ EdU DNA cell proliferation kit (RIBOBIO, Guangzhou, China), which measured cell proliferation by detecting the incorporation of the alkyne‐modified nucleoside EdU into DNA by copper‐catalyzed azide–alkyne click chemistry to attach fluorescent probes. Briefly, HNE2 and C666‐1 cells (1 × 10^4^ cells/96‐microwell plate) were seeded into 96‐well plates and treated with SM (6 μM) for 24 hr. After the medium was discarded, 50‐μM EdU was added for 2 hr at 37°C, then fixed in 4% PBS for 30 min, and permeabilized by 0.5% TritonX‐100 for 10 min. The cells were stained with 1 × Apollo reaction reagent for 30 min. All DNA contents of the cells were stained with Hoechst 33342 for another 30 min. Subsequently, pictures were obtained under 400 × magnifications under microscopy (Nikon, TI2‐E, Tokyo, Japan). At least five captured fields were randomly selected, and the EdU positive cells were calculated. The percentage of EdU positive cells = (EdU positive cells/Hoechst stain cells) × 100.

### 2.5 Colony formation assay

C666‐1 and HNE2 cells (5 × 10^2^ cells/6‐well plate) in logarithmic growth phase were seeded in 6‐well plates and treated with SM (4 μM) at 37°C in 5% humidified CO_2_ for 9 days with continuously changing RPMI 1640 medium supplemented with 10% FBS every 3 days. After that, the cells were fixed with 4% paraformaldehyde (Sigma‐Aldrich) and stained by 0.1% crystal violet (Sigma‐Aldrich) for duration of 30 min. Visible colonies from randomly selected five fields were manually counted under a microscope (Nikon, TI2‐E, Tokyo, Japan). The results were shown as the fold change relative to that of vehicle control group.

### 2.6 Western blot analysis

The detailed procedure was reported previously (Zhao et al., 2015). Protein concentrations were estimated using the Bio‐Rad protein assay kit (Hercules, CA, USA). Samples were resolved by 5 × SDS‐sample buffer and separated on SDS polyacrylamide gels. Each membrane was blocked in 5% (w/v) nonfat milk in Tris‐buffered saline with 0.1% (v/v) Tween‐20 for 1 hr followed by incubating with specific primary antibodies against SP1 and β‐actin (1:1000) at 4°C overnight. The membranes were washed and incubated with a secondary goat antibody raised against rabbit IgG conjugated to horseradish peroxidase (Cell Signaling Technology, Inc., Beverly, MA, USA) and were visualized using HRP‐conjugated anti‐rabbit secondary antibodies and enhanced chemiluminescence (Immobilon Western; Millpore, Billerica, MA, USA) followed by observing the signals under the Molecular Imager ChemiDoc XRS Gel Imagine System (Bio‐Rad, Hercules, CA, USA). Image software (National Institutes of Health, Bethesda, MD, USA) was applied to quantify and compare the intensity of single band between the control and proteins of interest.

### 2.7 Quantitative real‐time PCR

A quantitative real‐time PCR (qRT‐PCR) assay was developed for the detection and quantification of SP1, CCAT1, and miR7‐5p transcripts as described previously (Chen, Tang, Wu, Zheng, Yang & Hann, 2015). We used a qRT‐PCR assay to detecting the expressions of SP1, CCAT1, and miR7‐5p. Expression levels of SP1 and CCAT1 were normalized to that of the housekeeping gene GAPDH as the control. MiR7‐5p expression was defined based on Ct values, and relative expression levels were normalized according to the expression of small nuclear RNA U6. The sequences of primers are listed in Table.

Table The primer sequences of gene amplification by qRT‐PCR

**Table:** 

GAPDH	Forward sequence 5′‐CTCCTCCTGTTCGACAGTCAGC‐3′
	Reverse sequence 5′‐CCCAATACGACCAAATCCGTT‐3′
CCAT1	Forward sequence 5′‐GTGAGGACATGCAGCTTTCA ‐3′
	Reverse sequence 5′‐TGCTGCCT TTGATGTAGTCG‐3′
SP1	Forward sequence 5′‐ATTAACCTCAGTGCATTGGGTA‐3′
	Reverse sequence 5′‐AGGGCAGGCAAATTTCTTCTC‐3′
U6	Forward sequence 5′‐ATTGGAACGATACAGAGAAGATT‐3′
	Reverse sequence 5′‐GGAACGCTTCACGAATTTG‐3′
miR 7‐5p	Forward sequence 5′‐ACGTTGGAAGACTAGTGATTT‐3′
	Reverse sequence 5′‐TATGGTTGTTCTGCTCTCTGTCTC‐3′

Melting curves was applied to monitor nonspecific amplifications and the 2^‐ΔΔCt^ method was used to calculate relative expression changes of SP1, CCAT1, and miR7‐5p. Cells were treated with Trizol reagent for RNA extraction (Roche, Shanghai, China), and the first‐strand cDNA was synthesized from total RNA (1 μg) by reverse transcription using PrimeScript™RT Reagent Kit (GenePharma Co., Ltd. Shanghai, China) according to the instructions provided by manufacturer. QRT‐PCR was performed in a 20‐μl mixture containing 2 μl of the cDNA preparation and using ChamQ^TM^ Universal SYBR Green qPCR Master Mix (Vazyme Biotech Co., Ltd. Nanjing, Jiangsu, China) on an ABI 7500 real‐time PCR system (Applied Biosystems, Grand Island, NY, USA). The PCR conditions were as follows: 30 s at 95°C, followed by 40 cycles of 10 s at 95°C, and 30 s at 60°C. Each sample was tested in triplicate. Threshold values were determined for each sample/primer pair; the average and standard errors were calculated.

### 2.8 Transient transfection assay

This procedure was reported previously (Tang et al., 2015). Briefly, NPC cells (2.5 × 10^5^ cells/well) were seeded in 6‐well dishes and grown to 60–70% confluence. The mimics of miR7‐5p (100 nM) and the negative controls purchased from Ribo Biological Co., Ltd. (Guangzhou, China), control and SP1 overexpression vectors (pCMV6‐SP1; 0.8 μg/ml**),** MO2‐CCAT1 (0.8 μg/ml) **were transfected into the cells** using lipofectamine 3000 transfection reagent (Invitrogen, Shanghai, China) **for up to 30 hr based on the instruction from the provider, followed by treating with** SM **for an additional 24 or 48 hr** for other experiments. In the separated experiments, control and wild type SP1 promoter constructs (purchased from GeneCopoeia, Inc., Rockville, MD, USA) with or without 0.2 μg of the internal control secreted alkaline phosphatase were cotransfected into the cells with the lipofectamine 3000 transfection reagent. The preparation of cell extracts and measurement of luciferase activities were determined using Luc‐Pair™ dual‐luciferase HS assay kit (GeneCopoeia, Inc., Rockville, MD, USA).

### 2.9 Dual‐luciferase assay

The wild and mutation types of SP1 3'‐UTR luciferase vectors (0.6 μg/ml each) obtained from GeneCopoeia, Inc. (Rockville, MD, USA) were transfected into the cells with either miR7‐5p mimics (100 nM) or negative control using lipofectamine 3000 reagent; the preparation of cell lysis and measurement of luciferase activities were determined using the dual‐luciferase HS assay kit (GeneCopoeia). Luciferase activity was normalized with RLuc activity within each sample. In the separate experiments, SP1 promoter plasmids (0.6 μg/ml each) with Renilla luciferase reporters as an internal control were purchased from GeneCopoeia (Rockville, MD, USA). MO2‐CCAT1 (0.8 μg/ml**),** the control vector, and miR7‐5p mimics or negative controls, purchased from GeneCopoeia were cotransfected into the cells with the lipofectamine 3000. A luciferase activity assay was performed using the Luc‐Pair™ dual‐luciferase HS assay kit (GeneCopoeia) according to instructions from the manufacturer. Firefly luciferase activity was normalized to Renilla luciferase activity among every sample. Each experiment was repeated at least three times in triplicate.

### 2.10 Xenograft mouse model

Xenograft studies were conducted under the guidelines of the Institutional Animal Care Use Committee of Guangdong Provincial Hospital of Chinese Medicine with approved protocols (the Ethics Approval Number 2018067). A total of 33 female nude mice (6‐weeks‐old) were obtained from Beijing Vital River Laboratory Animal Technology Co., Ltd. (Beijing, China) and maintained at the Animal Center of Guangdong Provincial Hospital of Chinese Medicine in a specific pathogen‐free environment with food and water provided. C666‐1 cells carrying luciferase reporter gene (C666‐1‐Luc, obtained from the Guangzhou Land Biological Technology Co., Guangzhou, China; 1 × 10^6^ cells) were injected subcutaneously in nude mice. Mice were randomly divided into three groups (Con, low dose [3 mg/kg], and high doses [6 mg/kg] of SM), and xenografts were allowed to grow until tumors were detectable with calipers before treatment by intraperitoneal injection.

For bioluminescence imaging procedure, mice were anesthetized by inhalation of 2% isoflurane at the beginning and the end of treatment followed by injecting peritoneal with 150 mg/kg D‐luciferin in 200 μl (Xenogen; PerkinElmer, Waltham, MA, USA). The intensity of BLS in the luminescent area of the tumor was determined using the IVIS‐200 Imaging System (Xenogen, Alameda, CA, USA) and reported as photons/sec. Tumor volume was calculated using the formula for a spheroid: volume = (width^2^ × length) × 0.5. Body weights of mice were measured once a week. All mice were euthanized on 12 days for the collection of tumor samples. The corresponding extracted xenografts were processed for detecting the expression of SP1, CCAT1, and miR7‐5p by western blot and qRT‐PCR analysis, respectively.

### 2.11 Statistical analysis

All data were expressed as *M* ± *SD* of three independent experiments. Differences between groups were assessed by one‐way ANOVA and two‐tailed Student's *t*‐tests (GraphPadPrism5.0 software, LaJolla, CA, USA). The results in graphs were presented relative to the control. Asterisks shown in the figures indicate significant differences of experimental groups in comparison with the corresponding control condition (*p* < .05, see figure legends).

## 3 RESULTS

### 3.1 SM ‐nhibited growth of NPC cells

**Figure 1:**
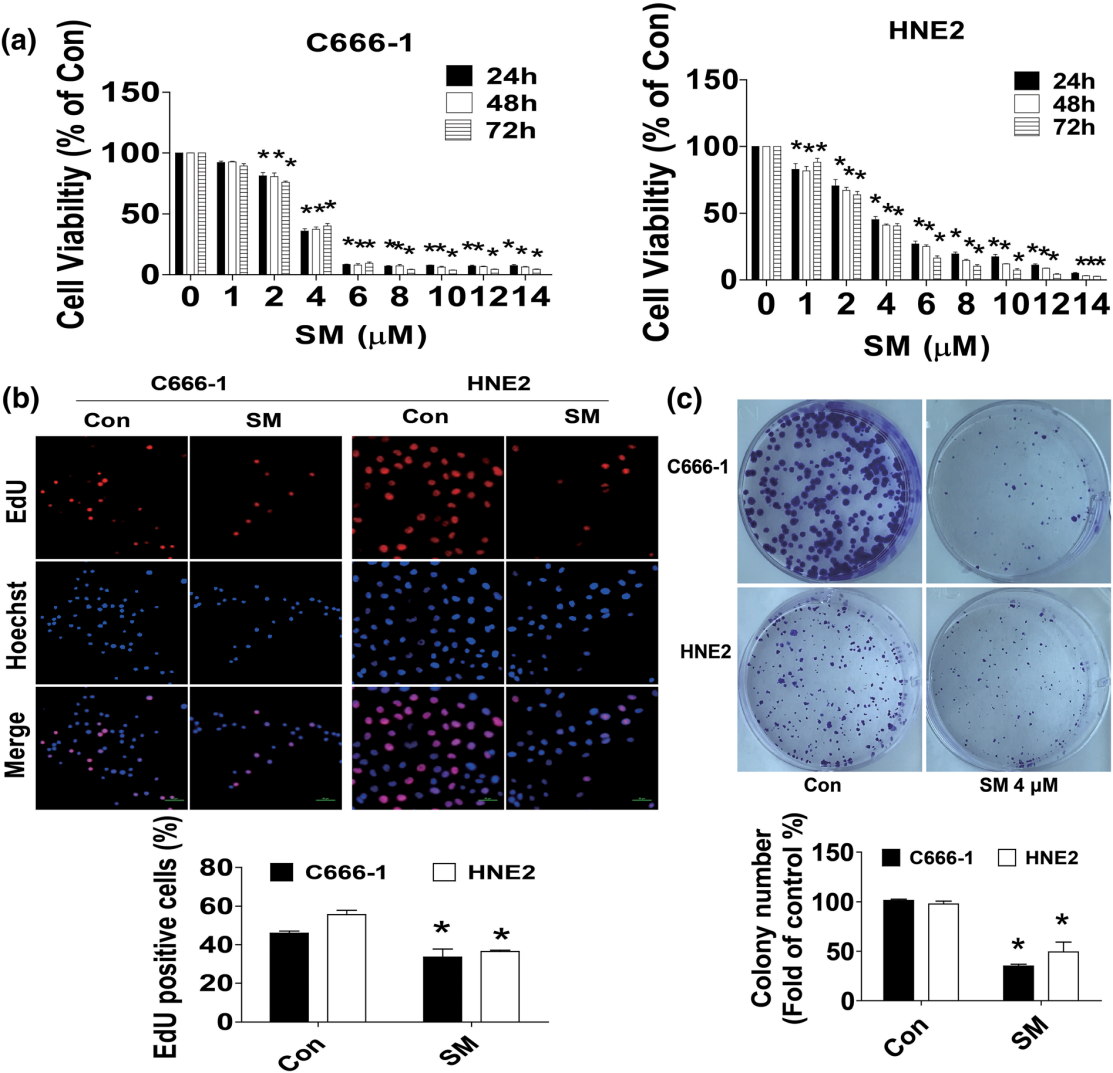
Solamargine (SM)‐inhibited growth of nasopharyngeal carcinoma cells. (a) HNE2 and C666‐1 cells were treated with increased doses of SM for up to 72 hr followed by measuring cell viability via MTT assay as described in Section 2. (b) HNE2 and C666‐1 cells were treated with SM (6 μM) for 48 hr, followed by determination of cell growth with the Cell‐Light^TM^ EdU DNA cell proliferation kit. The image was magnified 10×. Hoechst was used to stain all the nuclei. At least 5 captured fields were randomly selected, and the percentage of EdU positive cells = (EdU positive cells/Hoechst stain cells) × 100. Scale bars, 10 μm. (c) C666‐1 and HNE2 cells (5 × 10^2^ cells/6‐well plate) were seeded in 6‐well plates and treated with SM (4 μM) at 37°C in 5% humidified CO_2_ for up to 9 days. After that, the cells were fixed with 4% paraformaldehyde (Sigma‐Aldrich) and stained by 0.1% crystal violet, and the visible colonies were counted under a microscope as described in Section 2. *indicates significant difference as compared to the untreated control group (*p* < .05)

We initially started to examine the effect of SM on NPC cell growth. We showed that SM inhibited the growth of HNE2 and C666‐1 NPC cells in the dose‐dependent manner with significant effect most observed at 4–6 μM for up to 72 hr (Figure 1a). The IC50 was 3.207, 3.125, 3.126 and 3.273, 2.989, 2.853 μM in HNE2 and C666‐1 cells, respectively. Moreover, we further assessed the relative contribution to inhibition attributed to the SM using Cell‐Light^TM^ EdU DNA cell proliferation kit. We found that compared with the untreated control cells, SM also significant inhibited growth of NPC cells (Figure 1b). In addition, colony formation assays showed that SM significantly inhibited growth of HNE2 and C666‐1 cells (Figure 1c). Together, these findings suggested that SM significantly inhibited growth of NPC cells.

### 3.2 SM decreased lncRNA CCAT1 and increased miR7‐5p expression, and there was reciprocal interaction of CCAT1 and miR7‐5p

**Figure 2:**
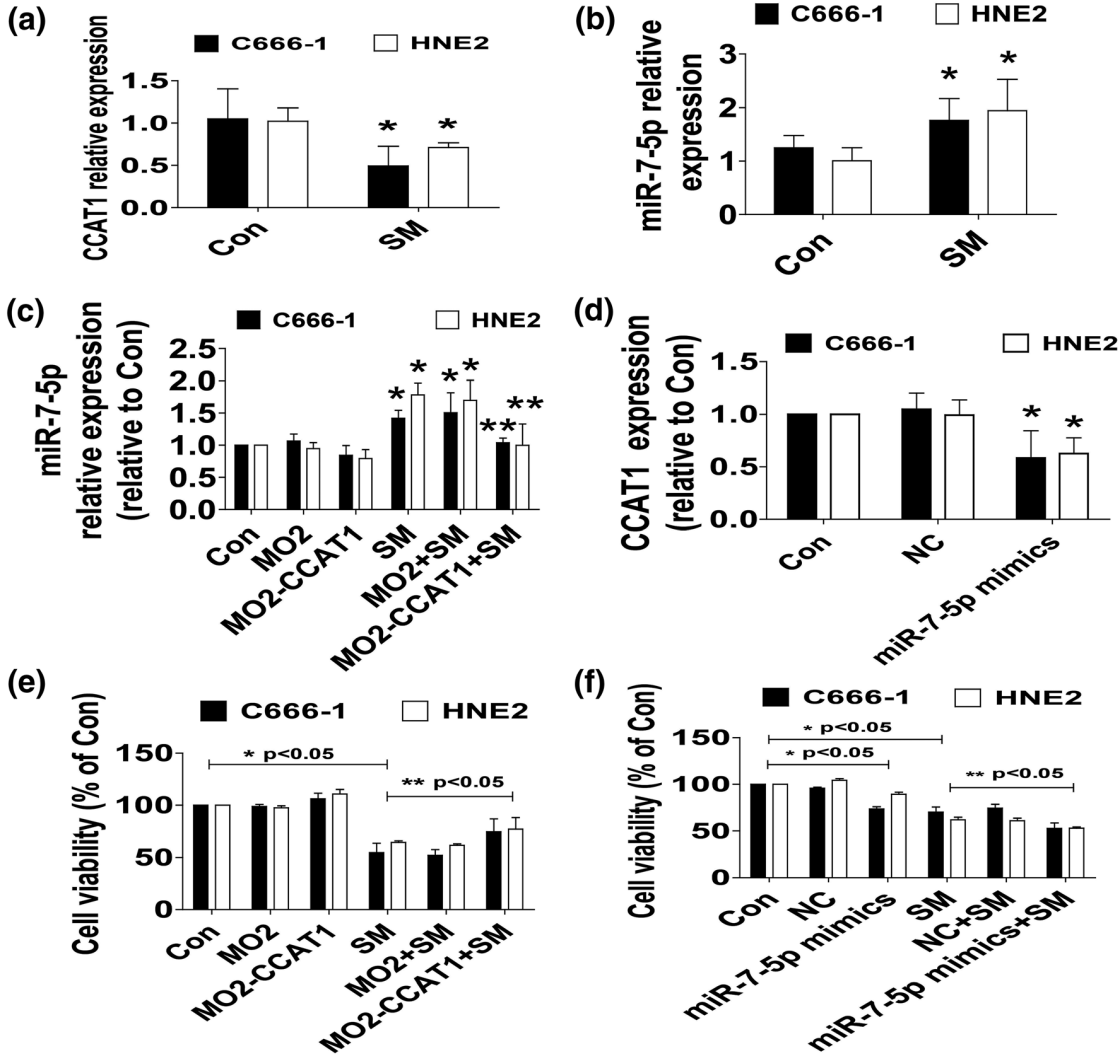
Solamargine (SM) decreased lncRNA CCAT1 and increased miR7‐5p expression, and there was reciprocal interaction of CCAT1 and miR7‐5p. (a–b) Total RNA were isolated from HNE2 and C666‐1 cells and processed for determining the levels of CCAT1 and miR7‐5p via qRT‐PCR. (c) HNE2 and C666‐1 cells were transfected with the control or CCAT1 expression vectors (0.8 μg/ml each) for up to 24 hr followed by exposure the cells to SM for an additional 24 hr, afterwards, miR7‐5p expressions were detected via qRT‐PCR. (d) HNE2 and C666‐1 cells were treated with the control or the miR7‐5p mimics (100 nM) for up to 48 hr followed by measuring CCAT1 expression via qRT‐PCR. (e–f) HNE2 and C666‐1 cells were transfected with the control or the CCAT1 expression vectors (0.8 μg/ml each) or treated with the control or the miR7‐5p mimics (100 nM) for 24 hr before exposure the cells to SM for an additional 24 hr followed by determining cell growth via MTT. Values and bar graphs are presented as the *M* ± *SD* of three independent experiments performed. *indicates significant difference from the control group (*p* < .05). **indicates significant difference from the SM alone (*p* < .05). CCAT1, colon cancer‐associated transcript‐1; NC, miR‐negative control

We then explored the molecular mechanisms that may be involved in the inhibitory response by SM in NPC cells. We first assessed the role of lncRNAs and miRNAs, such as CCAT1 and miR7‐5p, which were reported to be involved in the occurrence and development of cancers including NPC (Deng, Yang, Xu & Zhang, 2015; Dong, Yuan & Jin, 2018; Guo & Hua, 2017; He X, 2014; Kim, 2014; Nissan et al., 2012; Shan T, 2017). We showed that SM decreased lncRNA CCAT1 and increased miR7‐5p expressions in HNE2 and C666‐1 cells, respectively (Figure 2a,b). Moreover, we found that overexpressed CCAT1 overcame SM‐induced expression of miR7‐5p (Figure 2c). Conversely, the mimics of miR7‐5p inhibited CCAT1 expression in HNE2 and C666‐1 cells (Figure 2d). This indicated a potential interaction between inhibited CCAT1 and induced miR7‐5p. To further delineate the potential roles of CCAT1 and miR7‐5p in NPC cell growth inhibition, we showed that overexpressed CCAT1 overcame, whereas the mimics of miR7‐5p strengthened SM‐inhibited growth of HNE2 and C666‐1 cells (Figure 2e,f). Note that exogenously expressed CCAT1 alone showed some induced impact and the mimics of miR7‐5p alone significantly inhibited NPC cell proliferation (Figure 2e,f). Together, the above results indicated that the regulation and reciprocal interaction of CCAT1 and miR7‐5p contributed to the effect of SM on inhibition of NPC cell growth.

### 3.3 SM‐reduced protein, mRNA expression, and promoter activity of SP1, which were overcame by overexpressed CCAT1 but strengthened by the mimics of miR7‐5p

**Figure 3:**
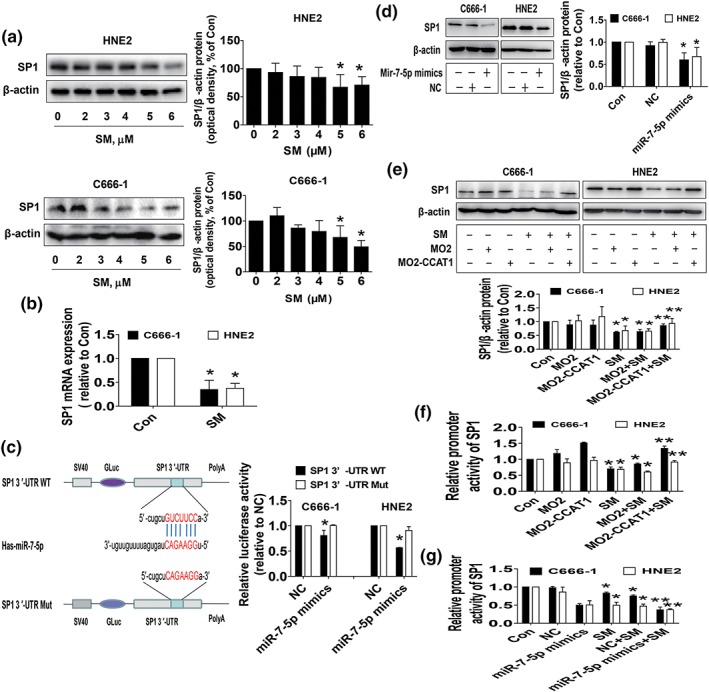
Solamargine (SM)‐reduced protein, mRNA expression, and promoter activity of SP1, which were overcame by overexpressed colon cancer‐associated transcript‐1 (CCAT1) but strengthened by the mimics of miR7‐5p. (a) HNE2 and C666‐1 cells were treated with the indicated doses of SM for 24 hr. Afterwards, the cell lysate was harvested and the protein expression of SP1 was measured by western blot. GAPDH was used as loading control. The figures are representative cropped gels/blots that have been run under the same experimental conditions. (b) Total RNA was isolated from the HNE2 and C666‐1 cells treated with SM for 24 hr and processed for determining the levels of SP1 via qRT‐PCR. (c) HNE2 and C666‐1 cells were transfected with the control and miR7‐5p mimics (100 nM) for 24 hr followed by measuring SP1 protein expression. (d) The luciferase reporter constructs containing a wild type and mutant SP1 sequences were shown (upper panel). HNE2 and C666‐1 cells were transfected with the SP1 3'‐UTR‐WT or SP1 3’‐UTR‐Mut vectors (0.8 μg/ml each) for 24 h, then treated with the miR7‐5p mimics (100 nM) or miR‐negative control (NC) for an additional 48 hr. Afterwards, the luciferase activity was detected using Luc‐Pair™ dual‐luciferase HS assay kit as described in Section 2 (lower panel). (e) HNE2 and C666‐1 cells transfected the control and CCAT1 expression vector (0.8 μg/ml each) for 24 hr, followed by treating with the SM (6 μM) for an additional 24 hr. Afterwards, the SP1 protein levels were determined by western blot. GAPDH was used as loading control. The figures are representative cropped gels/blots that have been run under the same experimental conditions. (f–g) HNE2 and C666‐1 cells were transfected with the control and CCAT1 expression vector (0.8 μg/ml each), miR7‐5p mimics (100 nM), or miR‐negative control (NC), the SP1 promoter construct ligated to luciferase reporter gene and an internal control secreted alkaline phosphatase for 24 hr before exposure of the cells to SM (6 μM) for an additional 24 hr. Afterwards, the SP1 promoter activity was detected using Luc‐Pair™ dual‐luciferase HS assay kit as described in Section 2. Values in bar graphs were given as the *M* ± *SD* from three independent experiments. *indicates significant difference as compared with the untreated control group (*p* < .05). **indicates significant difference from the SM alone (*p* < .05)

To gain insight into the molecular mechanisms by which SM inhibited NPC cell growth, we further search for the potential molecular targets that may link to CCAT1 and miR7‐5p, and cell growth inhibition. SP1 transcription factor has been reported to be involved in the variety of essential biological processes, such as cell growth, differentiation, apoptosis, and carcinogenesis (Vizcaino, Mansilla & Portugal, 2015). We also found that suppression of SP1 contributed to the inhibition of human lung cancer cell growth (Chen, Tang, Wu, Zheng, Yang & Hann, 2015). Herein, we showed that SM reduced the protein levels of SP1 in the dose‐dependent manner in HNE2 and C666‐1 cells (Figure 3a). SM also inhibited mRNA expression of SP1 as determined by qRT‐PCR in HNE2 and C666‐1 cells (Figure 3b). Next, we further interrogated the potential biological significance of the CCAT1 and miR7‐5p expressions on SP1 signaling that mediated the overall effects of SM. We showed that the mimics of miR7‐5p reduced protein levels and the luciferase activity in 3'‐UTR region of SP1 (Figure 3c,d). Of note, miR7‐5p–dependent inhibition of the SP1 3'‐UTR reporter activity was not observed in mutation of the predicted miRNA targeting sequences (Figure 3d). Interestingly, exogenously expression of CCAT1 reversed SM‐inhibited SP1 protein expression and promoter activity (Figure 3e,f). Moreover, as expected, the mimics of miR7‐5p strengthened SM‐inhibited SP1 promoter activity (Figure 3g). Together, these findings suggested that repression of SP1 and CCAT1, and more importantly, the regulation and reciprocal interactions of CCAT1 and miR7‐5p on SP1 at transcriptional and translational levels were involved in the anti‐NPC cell growth of SM.

### 3.4 While overexpression of SP1 had no effect on CCAT1, it attenuated the effect of SM on miR7‐5p expression and cell growth inhibition

**Figure 4:**
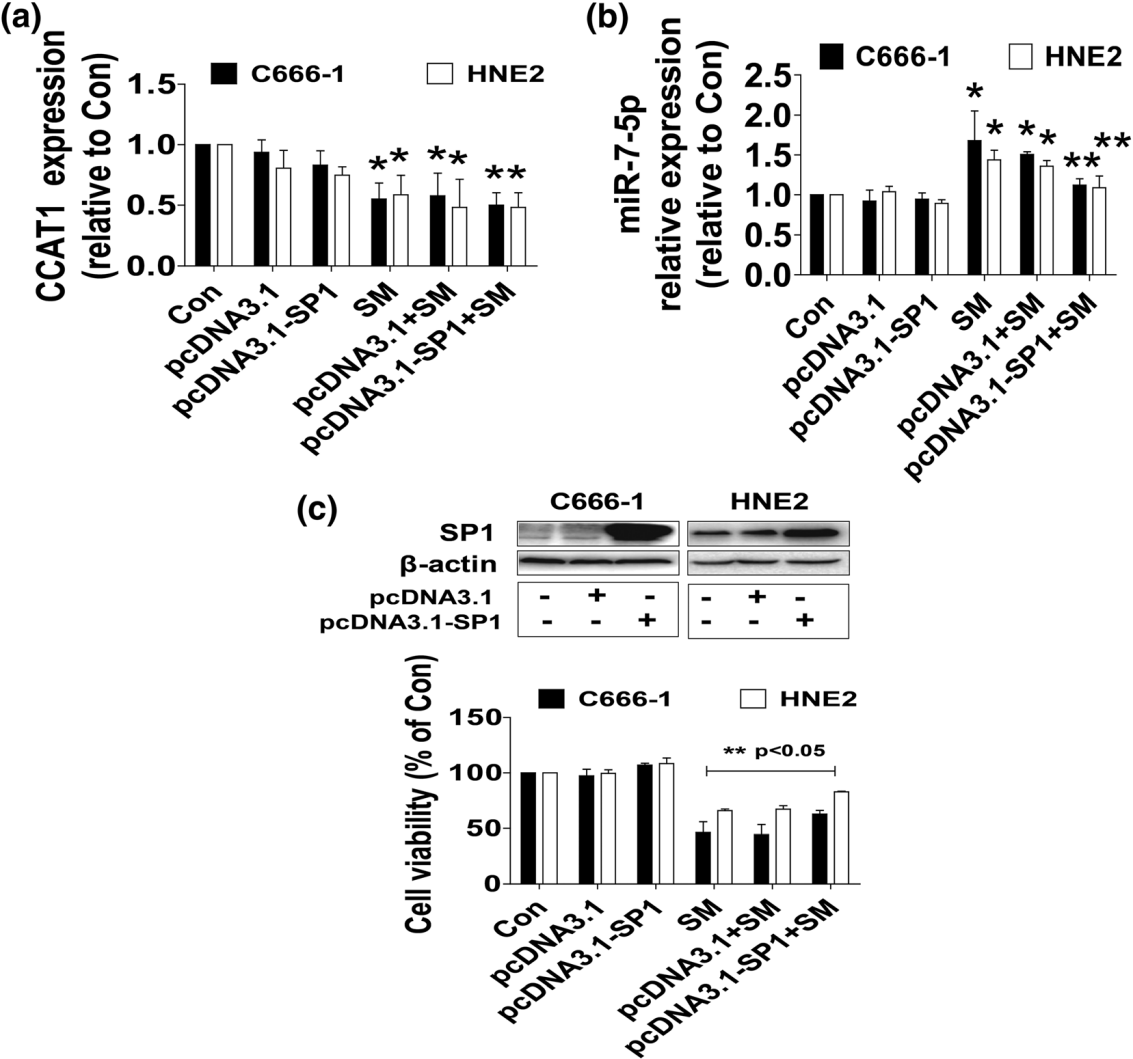
While overexpression of SP1 had no effect on CCAT1, it attenuated the effect of SM on miR7‐5p expression and cell growth inhibition. (a–b) Total RNA was isolated from the HNE2 and C666‐1 cells transfected with the control and SP1 expression vector for 24 hr before exposure the cells to SM for an additional 24 hr, followed by measuring CCAT1 and miR7‐5p expressions via qRT‐PCR. (c) HNE2 and C666‐1 cells were transfected with the control and SP1 expression vector for 24 hr before exposure the cells to SM for an additional 24 hr, followed by measuring cell viability via MTT assays described in Section 2. *indicates significant difference as compared with the untreated control group (*p* < .05). **indicates significant difference from the SM alone (*p* < .05). CCAT1, colon cancer‐associated transcript‐1; SM, solamargine

To further characterize the role and explore the functional relevance of SP1 expression changes following regulation of CCAT1 and miR7‐5p by SM, we transfected the exogenously expressed SP1 plasmid into the cells and found that, although overexpression of SP1 had no effect on SM‐reduced CCAT1 expression (Figure 4a), it reversed the effect of SM on miR7‐5p expression and cell growth inhibition in HNE2 and C666‐1 cells (Figure 4b,c). This result indicated the critical role of SP1 in regulation of cell growth in this process.

### 3.5 Effect of SM on tumor growth in vivo

**Figure 5:**
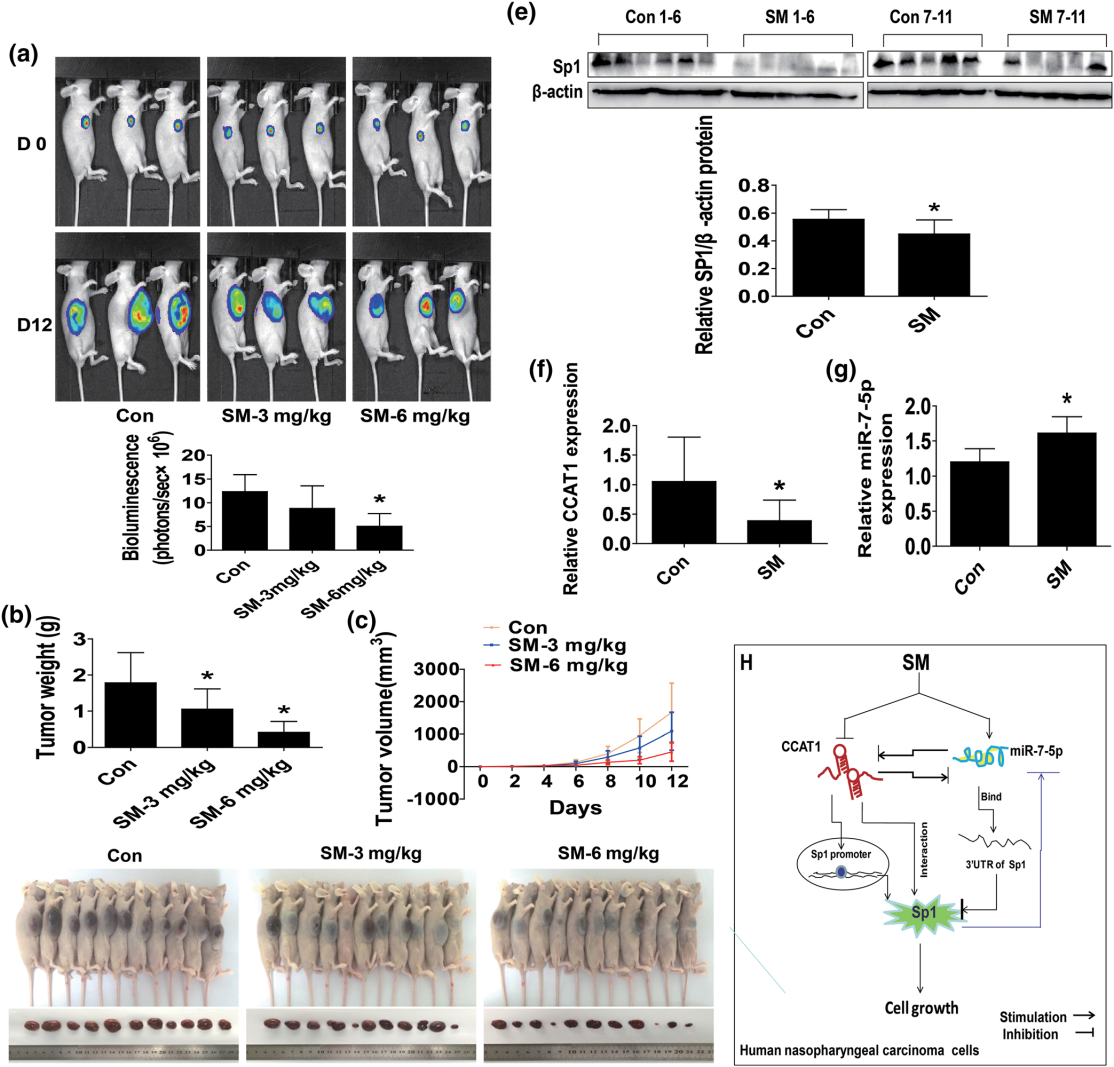
Effect of SM on tumor growth in vivo Mice (*n* = 10/group) were divided to three groups [con (saline), low (L, 3 mg/kg), and high (H, 6 mg/kg) doses], and SM was given around the 4th day after tumor cells injection by intraperitoneal injection for up to 12 days. (a) The xenografts were assessed by in vivo bioluminescence imaging at the end of the experiments (on Day 12). The tumor growth was monitored by injecting luciferin in the mice followed by measuring bioluminescence using IVIS imaging system. Imaging and quantification of signals were controlled by the acquisition and analysis software living image as described in Section 2. Representative images are shown. (b–c) The xenografts were harvested on Day 12, and then the volume (b) and weight (c) of tumors were measured. (d) The photographs of the SM alone and the vehicle‐treated xenografts derived from nude mice are shown. (e–g) At the end of the experiments, xenograft tumors were isolated from individual animals and the corresponding lysates were processed for detecting SP1, CCAT1, and miR7‐5p by western blot and qRT‐PCR, respectively. GAPDH was used as loading control. The bar graphs represented the tumor weight and volume of mice results of as *M* ± *SD*. *indicates the significant difference from untreated control (*p* < .05). (h) The diagram shows that SM inhibits the growth of NPC cells through reciprocal regulation of CCAT1 and miR7‐5p followed by inhibition of SP1 gene expression in vitro and in vivo. The intercorrelation among CCAT1, miR7‐5p and SP1, and the feedback regulatory loop unveil the novel molecular mechanism underlying the overall responses of SM in anti‐NPC. CCAT1, colon cancer‐associated transcript‐1; SM, solamargine

We also tested the effect of SM in tumor growth and expression of CCAT1 and miR7‐5p, and SP1 in xenograft mouse model. Luciferase‐expressing C666‐1 cells were injected subcutaneously into nude mice. Mice bearing xenografted tumor was treated by intraperitoneal injection once every other day for different doses of SM (3 and 6 mg/kg, respectively) for up to 12 days. We found that, compared with the control group, the two different doses of SM‐treated mice showed a significant delayed tumor growth, without any severe adverse events, as assessed by the Xenogen IVIS200 system (http://www.ncbi.nlm.nih.gov/pmc/articles/PMC4247304/figure/f1-etm-09-01-0162/ 5a). The differences in the levels of luciferase expression correlates with the tumor area. In addition, we noticed a significant reduction of the tumor weight in the SM treatment groups and sizes in the high dose of SM group as compared with the control one (Figure 5b–d). By western blot, fresh tumors harvested from the aforementioned experiment showed that SM efficiently decreased CCAT1 and SP1, while inducing miR7‐5p expressions in vivo in the high dose of SM treatment group as compared with that in the control one (Figure 5e–g).

## 4 DISCUSSION

Although NPC may be regarded as a rare cancer globally, it is notable for its high incident rate in special geographic and ethnic populations. In spite of the improvement and extensive application of the intensity modulated radiotherapy, image guided radiotherapy techniques, and other new therapeutic strategies, the recurrence and metastasis are still challenge for patient survival Therefore, identification of highly effective and low toxicity new adjuvant therapeutic agents to supplement current therapeutic modalities becomes a research focus. SM is a promising anticancer agent for various cancer types with mechanistic involvement of multiple pathways and molecular targets (Cui, Wen, Cui, Gao, Sun & Lou, 2012; Friedman, 2015; Munari et al., 2014; Zhou et al., 2014). There were so far no single studies demonstrating the effects of SM on NPC cells. In this study, we demonstrated significant inhibitory effects to NPC cells by SM. Our results indicated that SM inhibited the growth of NPC cells through reciprocal regulation of CCAT1 and miR7‐5p, followed by inhibition of SP1 gene expression in vitro and in vivo.

To further explore the potential mechanism, we explored the functional relevance of potential gene expression changes that involved in the inhibition of proliferation of NPC cell by SM. We tested the role of CCAT1 and miR7‐5p In NPC cells, CCAT1 was highly expressed NPC tissues compared with normal nasopharyngeal epithelial ones and silencing of CCAT1 inhibited growth, migration, and invasion in NPC cells (Dong, Yuan & Jin, 2018). Conversely, increased CCAT1 expression resulted in significant enhancing paclitaxel resistance in NPC cells (Wang, Zhang & Hao, 2017). Our results provided evidence for the regulation of CCAT1 by SM and implied the critical role of CCAT1 expression involved in the inhibitory effect of SM on NPC cell growth, and suggesting that CCAT1 could be a potential target of SM in the treatment of this type of malignancy. Also, our finding also demonstrated a vital role of miR7‐5p. MiR7‐5p has been recognized as a tumor suppressor involved in multiple cancers (Hu et al., 2019; Jia et al., 2019; Li, Qiu, An, Huang & Gong, 2018). Our data suggested that induction of miR7‐5p mediated the inhibitory effect of SM on NPC cell growth suggesting that miR7‐5p is a potential molecular target for the treatment of NPC. Moreover, there was a reciprocal interaction between the CCAT1 and miR7‐5p that may contribute to overall anti‐NPC effects of SM. The interregulation of CCAT1 and miRNAs including miR7 has been studies in different cancers including NPC (Wang, Zhang & Hao, 2017; Zhang et al., 2017a; Zhang, Cai, Jiang & Xu, 2019); CCAT1 increased cancer cell proliferation and metastasis by acting as a competing endogenous RNA to sponging miRNAs resulting in regulation of gene expression or through regulation of other signaling regulatory axis (Xu et al., 2018; Zhang, Cai, Jiang & Xu, 2019). Bioinformatics analysis and luciferase reporter experiments showed that several lncRNAs were direct target of miR7‐5p that were involved in the tumor growth, migration, invasion, and chemoresistance in several cancer types (Dong, Xie & Xu, 2019; Jia et al., 2019; Lai, Yang, Zhu, Ruan, Huang & Zhang, 2019; Mo, Liu, Li & Cui, 2019). One study showed that silencing of CCAT1 significantly inhibited the migration, invasion, and reversed EMT phenotype of intrahepatic cholangiocarcinoma cells through directly binding to miR‐152 (Zhang et al., 2017b). Of note, there was no report demonstrating a direct links of CCAT1 and miR7‐5p, thus, the detailed mechanism of this interaction required to be elucidated. Regardless, these aforementioned data provide potent mechanism for the regulation and interaction of lncRNA and miRNA, such as CCAT1 and miR7‐5p, in regulation of gene expression and cancer growth and progression, all of which may indicate promising biomarkers and potential therapeutic targets for the treatment of human cancer.

Moreover, our result also indicated important roles of SP1, a well‐known transcription factor that has been shown to be involved in the proof of concept functions in cancer biology. Our data suggested that reduction of SP1, and more importantly the interregulations of CCAT1, miR7‐5p and SP1, and the feedback regulatory loops of SP1 contributed to the inhibition of cell growth by SM. SP1 play major roles in the pathogenesis of various cancers. Consistent with this, one study showed that silencing of SP1 inhibited growth and migration and attenuated the effect of miR‐382 inhibitor on colorectal cancer cell behaviors (Ren, Zhang & Jiang, 2018). Also, SP1‐induced lncRNA terminal differentiation‐induced ncRNA (TINCR) was significantly increased in colorectal cancer tissues and cells. TINCR could act as a miR7‐5p sponge and silencing of TINCR suppressed proliferation, migration, and invasion in colorectal cancer cells (Yu et al., 2019). LncRNA ZNFX1 antisense RNA1 (ZFAS1) inhibited proliferation, migration, and development of chemoresistance in via targeting miR‐150‐5p, subsequently inhibited expression of SP1 as determined by luciferase assays, revealing a critical role of ZFAS1/miR‐150‐5p/SP1 axis in promoting proliferation, migration, and development of chemoresistance in epithelial ovarian cancer cells (Xia et al., 2017). Nevertheless, more experiments are required to determine if there is a physical binding between CCAT1, miR7‐5p, and SP1, and whether CCAT1 directly bind and sequester miR7‐5p from the target gene SP1 that influence SM‐inhibited NPC cell growth.

More importantly, our in vivo data were consistent with the findings from that in vitro, confirming the effect of SM on NPC growth inhibition and regulation of CCAT1, miR7‐5p, and SP1 expressions. We believed that the SM doses used in this study were reasonable and feasible which based on our and other studies (Chen, Tang, Xiao, Yang & Hann, 2017; Fu et al., 2019; Tang, Zheng, Wu, Xiao, Li & Hann, 2017). Regardless, more experiments and statistical analysis are required to further determine the critical role and correlations of CCAT1, miR7‐5p and SP1, and tumor sizes in this process in mice model. In addition, whether SM has potential in prolonging the survival and inhibiting metastasis in NPC xenografted tumors required to be elucidated.

In conclusion, our results show that SM inhibits the growth of NPC cells through reciprocal regulation of CCAT1 and miR7‐5p, followed by inhibition of SP1 gene expression in vitro and in vivo. The intercorrelations and regulations among CCAT1, miR7‐5p and SP1, and the feedback regulatory loop unveil the novel molecular mechanism underlying the overall responses of SM in anti‐NPC (Figure 5h).

## CONFLICT OF INTEREST

The authors declare that they have no competing interests.

## AUTHOR CONTRIBUTIONS

S.S.H. is fully responsible for the study designing, experiment adjustment, and drafting the manuscript. J.J.W. and X.J.T. performed most of the experiments involved. C.J.M. carried out transfection assays and some protein measurement by western blot and statistical analysis. Y.S. participated in coordination manuscript. WYW coordinated and provided important suggestionsincluding some reagents, along with critical reading of the paper. All authors read and approved the final manuscript.
